# mEPE-score: a comprehensive grading system for predicting pathologic extraprostatic extension of prostate cancer at multiparametric magnetic resonance imaging

**DOI:** 10.1007/s00330-022-08595-9

**Published:** 2022-03-15

**Authors:** Marco Gatti, Riccardo Faletti, Francesco Gentile, Enrico Soncin, Giorgio Calleris, Alberto Fornari, Marco Oderda, Alessandro Serafini, Giulio Antonino Strazzarino, Elena Vissio, Laura Bergamasco, Stefano Cirillo, Mauro Giulio Papotti, Paolo Gontero, Paolo Fonio

**Affiliations:** 1 Department of Surgical Sciences, Radiology Unit, University of Turin, Via Genova 3, 10126 Turin, Italy; 2 Urology Unit, Department of Surgical Sciences, University of Turin, Turin, Italy; 3 Radiology Unit, Mauriziano Umberto I Hospital, 10128 Turin, Italy; 4 Pathology Unit, Department of Medical Sciences, University of Turin, Turin, Italy; 5 Department of Medical Sciences, University of Turin, Turin, Italy

**Keywords:** Multiparametric magnetic resonance imaging, Prostatic neoplasms, Prostatectomy, Neoplasm staging, Scoring methods

## Abstract

**Objective:**

To investigate the diagnostic accuracy of the PI-RADS v2.1 multiparametric magnetic resonance imaging (mpMRI) features in predicting extraprostatic extension (mEPE) of prostate cancer (PCa), as well as to develop and validate a comprehensive mpMRI-derived score (mEPE-score).

**Methods:**

We retrospectively reviewed all consecutive patients admitted to two institutions for radical prostatectomy for PCa with available records of mpMRI performed between January 2015 and December 2020. Data from one institution was used for investigating diagnostic performance of each mEPE feature using radical prostatectomy specimens as benchmark. The results were implemented in a mEPE-score as follows: no mEPE features: 1; capsular abutment: 2; irregular or spiculated margin: 3; bulging prostatic contour, or asymmetry of the neurovascular bundles, or tumor-capsule interface > 1.0 cm: 4; ≥ 2 of the previous three parameters or measurable extraprostatic disease: 5. The performance of mEPE features was evaluated using the five diagnostic parameters and ROC curve analysis.

**Results:**

Two-hundred patients were enrolled at site 1 and 76 at site 2. mEPE features had poor sensitivities ranging from 0.08 (0.00–0.15) to 0.71 (0.59–0.83), whereas specificity ranged from 0.68 (0.58–0.79) to 1.00. mEPE-score showed excellent discriminating ability (AUC > 0.8) and sensitivity = 0.82 and specificity = 0.77 with a threshold of 3. mEPE-score had AUC comparable to ESUR-score (*p* = 0.59 internal validation; *p* = 0.82 external validation), higher than or comparable to mEPE-grade (*p *= 0.04 internal validation; *p *= 0.58 external validation), and higher than early-and-late-EPE (*p *< 0.0001 internal and external validation). There were no significant differences between readers having different expertise with EPE-score (*p *= 0.32) or mEPE-grade (*p *= 0.45), but there were significant differences for ESUR-score (*p *= 0.02) and early-versus-late-EPE (*p *= 0.03).

**Conclusions:**

The individual mEPE features have low sensitivity and high specificity. The use of mEPE-score allows for consistent and reliable assessment for pathologic EPE.

**Key Points:**

*• Individual PI-RADS v2.1 mpMRI features had poor sensitivities ranging from 0.08 (0.00–0.15) to 0.71 (0.59–0.83), whereas Sp ranged from 0.68 (0.58–0.79) to 1.00.*

*• mEPE-score is an all-inclusive score for the assessment of pEPE with excellent discriminating ability (i.e., AUC > 0.8) and Se = 0.82, Sp = 0.77, PPV = 0.74, and NPV = 0.84 with a threshold of 3.*

*• The diagnostic performance of the expert reader and beginner reader with pEPE-score was comparable (p = 0.32).*

**Supplementary Information:**

The online version contains supplementary material available at 10.1007/s00330-022-08595-9.

## Introduction

Multiparametric magnetic resonance imaging (mpMRI) is playing an increasingly important role in the diagnostic pathway of patients with prostate cancer (PCa), both in identifying suspicious lesions and in locoregional staging [1].

Correct tumor staging is critical for disease management. Curative treatment becomes more likely in the absence of pathologic extra prostatic extension (pEPE); additionally, organ-confined disease allows for more conservative surgical approaches [2, 3].

Many studies investigated the accuracy of mpMRI in local staging [4]. Use of magnetic field strengths, endorectal coils, and combinations of anatomic and functional mpMRI methods differs between studies, including also emerging technologies based on radiomics or artificial intelligence [5–7]. A recent meta-analysis [4] reported that mpMRI has poor and heterogeneous sensitivity (Se) for local PCa staging, but high specificity (Sp). These disparate findings have driven the ongoing discussion regarding the use of mpMRI for PCa staging, as well as which mpMRI criteria are most accurate and how to combine them.

In recent years, a few imaging-based scores were proposed for the assessment of EPE at mpMRI (mEPE). Among them, a score based on qualitative parameters (i.e., Likert scale) showed good performance [8, 9] and low inter-observer variability [8]; however, Likert scales lack objective criteria and are often difficult to reproduce. Three more “quantitative” scores [10–12] attempted to create a progressive risk score by combining in different ways some of the imaging features present in Prostate Imaging Reporting and Data System (PI-RADS) v2.1 [1], i.e., “asymmetry or invasion of the neurovascular bundles, bulging prostatic contour, irregular or spiculated margin, obliteration of the recto-prostatic angle, tumor-capsule interface greater than 1.0 cm, breach of the capsule with evidence of direct tumor extension or bladder wall invasion.”

To the best of our knowledge, the literature lacks studies that examine individually the PI-RADS v2.1 features considered EPE predictors, or that employ an all-inclusive score that incorporates all of them. The aim of the study was to investigate the diagnostic accuracy of the PI-RADS v2.1 mEPE features in predicting pEPE of PCa, as well as to develop and validate a comprehensive mEPE-score.

## Methods

The study was piloted in agreement with the 1964 Helsinki Declaration and its later amendments. The Local Institutional Review Board of each Institution approved this retrospective study and waived the need for written informed consent.

### Study design and patient population

The study involved patients with a biopsy-proven PCa and pre-operative mpMRI examination performed in one of two different structures: Azienda Ospedaliero-Universitaria Città della Salute e della Scienza di Torino - Presidio Ospedaliero Molinette, hereon identified as site 1, and Azienda Ospedaliera Ordine Mauriziano di Torino - Ospedale Umberto I, hereon identified as site 2.

In both sites, all consecutive patients admitted between January 2015 and December 2020 were retrospectively reviewed.

We design a study aimed at:

1. Evaluating the diagnostic value of each mEPE parameter in the PI-RADS v2.1 criteria [1] for pEPE;
2. Introducing a standardized grading system including all such variables;
3. Comparing its performance against the three mEPE quantitative scoring systems in the literature [10–12];
4. Validating internally and externally the mEPE-score;
5. Investigating its performance for different levels of biopsy ISUP and of expertise of observers.

Inclusion criteria for enrolment were:

1. Radical prostatectomy for PCa.
2. Definitive histological examination based on International Society of Urological Pathology criteria [13–16].
3. Pre-operative mpMRI examination performed no earlier than 6 months from the date of radical prostatectomy.

Exclusion criteria were:

1. Hormonotherapy
2. Radiotherapy
3. Previous endoscopic prostate resection
4. Significant artifacts (i.e., motion artifact, artifactual distortion caused by air and/or stool in the rectum, metallic artifact caused by hip prosthesis replacement) at mpMRI, Prostate Imaging Quality (PI-QUAL) score < 3 [17], or incomplete exams (i.e., interrupted for claustrophobia).

**Fig. 1:**
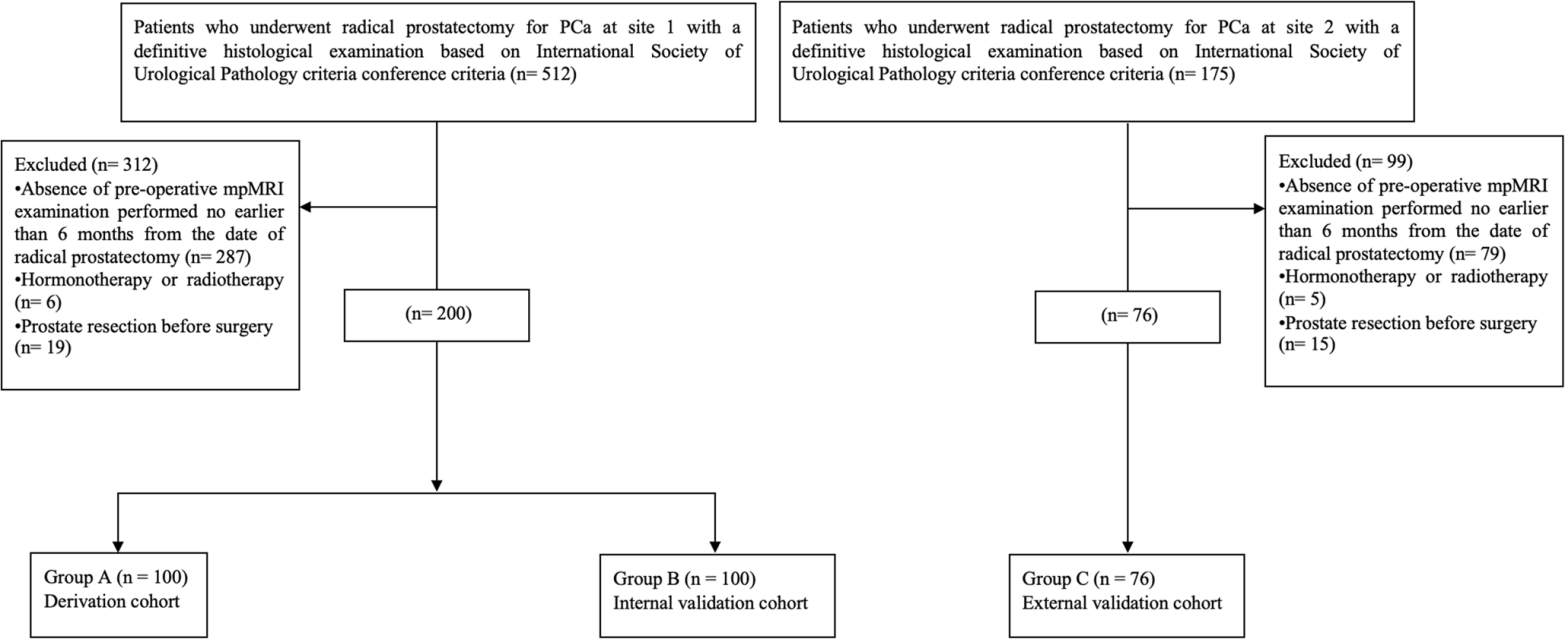
Flow diagram

The flow diagram is reported in Fig. 1.

### mpMRI protocol

The MR examination was performed at 1.5 T (Achieva and Ingenia, Philips Medical Systems) using a 32-phased-array coil. A minimal preparation enema was administered to the patient in the hours prior to the examination. If tolerated, a 1 mg hyoscinbutylbromide (Buscopan, Boehringer Ingelheim) intravenous injection was given to the patient to reduce peristaltic motion. The MR protocol is shown in Tables S1 and S2.

### Pathology

All histopathological examinations were performed by three senior pathologists (L.D., L.M., and D.P.), all of whom had more than 10 years of experience in genitourinary pathology. The processing and reporting were carried out in accordance with the International Society of Urological Pathology (ISUP) recommendations [13–16]. Notably, the prostate margins were inked, the prostate and seminal vesicles were completely embedded and examined using whole mounts and standard blocks, and the presence and location (i.e., side) of pEPE were included in the pathological report.

### Imaging analysis

Image evaluation was performed using a dedicated medical imaging workstation (IntelliSpace Portal, Philips Healthcare).

Image analysis at site 1 was done in consensus by an expert radiologist (R.F.; > 2000 cases analyzed) and a beginner radiologist (M.G.; about 500 cases). Image analysis at site 2 was done in double-blinded fashion, by an expert radiologist (S.C., > 4000 cases analyzed) and a beginner radiologist (A.F., > 500 cases) [18]. In both sites, the radiologists were blinded to the pathologic data.

**Fig. 2:**
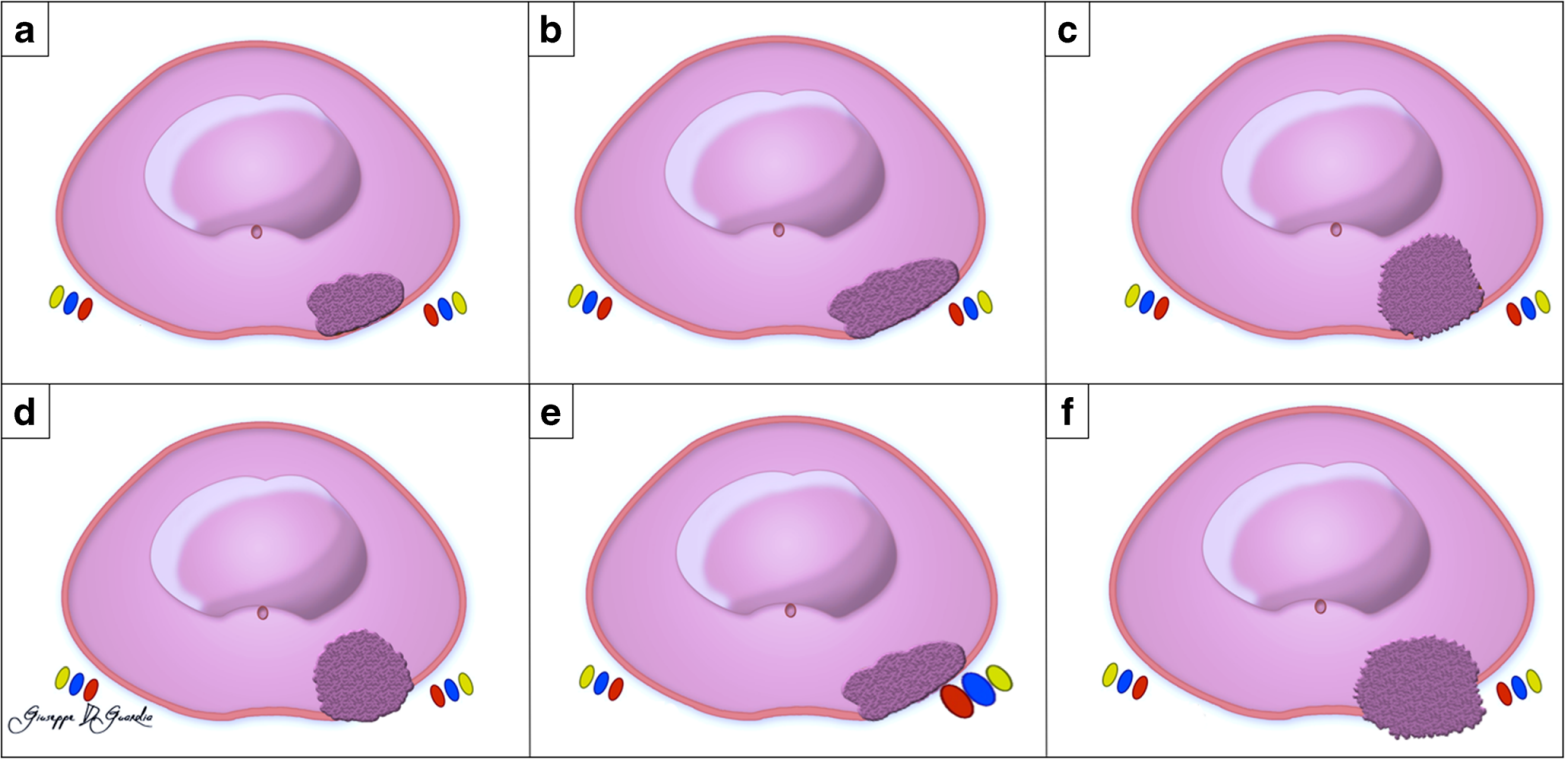
Schematic representation of mpMRI features for predicting pathologic EPE. Abutment of the capsule (**a**), tumor-capsule interface > 1.0 cm (**b**), irregular margin (**c**), bulging prostatic contour (**d**), asymmetry of the neurovascular bundles (**e**), breach of the capsule with evidence of direct tumor extension or bladder wall invasion (**f**)

The index lesion was defined as the largest suspected tumor focus or, alternatively, the most likely to contribute to mEPE. Each lesion was assigned a sector map location, and the mEPE features (Fig. 2) present in PI-RADS v2.1 were reported [1].

Following the radiological evaluation, a beginner radiologist (F.G. with approximately 500 cases analyzed) who was not involved in the imaging analysis but had access to all available data from mpMRI and pathology reviewed the pathology reports to see if pEPE was found on radical prostatectomy specimens at the site of the index lesion.

### Statistical analysis

Continuous variables are expressed as median (1st–3rd quartile), and categorical variables as counts and percentages. The investigation for significant association with pEPE used non-parametric tests (Mann-Whitney’s for independent continuous variables and Fisher’s exact test for dichotomic ones) and binary logistic regression (BLR). The concordance between two individual items of two distributions, including scores by two readers, was measured by Cohen’s kappa coefficient (0 ≤ *k* ≤ 1). The following labels are assigned to the corresponding ranges of kappa: < 0.00: poor, 0.00–0.20: slight; 0.21–0.40: fair; 0.41–0.60: moderate; 0.61–0.80: substantial; and 0.81–1.00: almost perfect [19].

The performance of the scoring approaches was evaluated with the ROC curve (plot of Se vs. (1-Sp)) and quantified by the area under the curve (AUC). The threshold for positive diagnosis was set at the value which satisfied the triple condition of (i) maximization of the harmonic mean of Se and Sp, (ii) maximization of Youden’s index (Se+Sp-1), and (iii) minimization of the distance of the curve from the upper left corner (Se = Sp = 1).

Significant association corresponds to *p* < 0.05 and 95%CI of BLR odds ratio not including 1. Analyses were performed using StatPlus for Macintosh ver. 7 (AnalystSoft, Walnut).

## Results

The patients who met the inclusion criteria were 200 at site 1 and 76 at site 2. No patients were excluded for technically limited study (i.e., all studies were PI-QUAL score > 3). The clinical data, reported in Table 1, showed no significant differences between sites.

**Table 1:** Clinical data of enrolled patients

	Site 1 (*N *= 200)	Site 2 (*N* = 76)	*p*
Age (years)	66 (60–71)	67 (62–70)	0.79
PSA (ng/mL)	7 (5–10)	7 (6–10)	0.59
ISUP grade at radical prostatectomy			0.34
1	1 (0.5%)	2 (3%)	
2	83 (41.5%)	41 (54%)	
3	77 (38.5%)	26 (34%)	
4	24 (12%)	6 (8%)	
5	1 (0.5%)	2 (3%)	
ISUP grade at radical prostatectomy ≥ 3	116 (58%)	34 (44%)	0.14
Pathologic EPE	85 (42.5%)	37 (48.7%)	0.43

*PSA*, prostate specific antigen; *ISUP*, International Society of Urological Pathology; *EPE*, extraprostatic extension

The 200 patients at site 1 were split by alphabetical order into two groups, A and B, each composed by 100 patients. Since there were no significant differences in the features of the two subgroups (see Table 2), group A was randomly picked for deriving the new comprehensive score, assigning to Group B the task of internal validation.

**Table 2:** Clinical and radiological data

	Group A	Group B	*p*
Age (years)	66 (60–71)	67 (61–72)	0.59
PSA (ng/mL)	7.0 (4.8–9.6)	7.2 (5.15–9.95)	0.49
ISUP grade at radical prostatectomy			0.46
1	1	0	
2	39	45	
3	37	40	
4	15	9	
5	9	6	
ISUP grade at radical prostatectomy ≥ 3	61	55	0.47
Pathologic EPE	45	41	0.67
Capsular abutment	97	93	0.33
Tumor-capsule interface (mm)	8 (5–11)	8 (5–12)	0.88
Tumor-capsule interface > 10 mm	31	34	0.76
Irregular or spiculated margin	53	55	0.89
Bulging prostatic contour	37	35	0.88
Asymmetry of neurovascular bundle	13	12	≥ 0.99
Measurable extraprostatic disease	9	11	0.8
Obliteration of recto-prostatic angle	4	6	0.75

*PSA*, prostate specific antigen; *ISUP*, International Society of Urological Pathology; *EPE*, extraprostatic extension

### Determination of EPE-score

The diagnostic performance of the individual mEPE features included in PI-RADS v2.1 was investigated using as benchmark the identification of pEPE on radical prostatectomy specimens at the location of the index lesion. For each feature, we computed detection rate (ratio of number of patients with pEPE to number of patients with the mEPE feature), Se, Sp, positive predictive value (PPV), and negative predictive value (NPV) and used univariate BLR to determine its association with pEPE. The results are summarized in Table 3: the feature with the lowest detection rate and highest Se was capsular abutment, followed by irregular or spiculated margin and bulging prostatic contour; the parameters with 100% detection rate and highest Sp (and lowest Se) were measurable extraprostatic disease and obliteration of recto-prostatic angle. For capsular abutment, measurable extraprostatic disease, and obliteration of recto-prostatic angle, the BLR odds ratio intervals were undetermined (0–infinite).

**Table 3:** Diagnostic features of individual mEPE features included in PI-RADS v2.1

		Diagnostic parameters				Univariate BLR	
	Detection rate	Sensitivity	Specificity	PPV	NPV	*p*	Odds ratio
Capsular abutment	45/97 (46%)	1	0.07 (0.004–0.14)	0.46	1	0.98	n.d.
Tumor-capsule interface > 10 mm	23/31 (74%)	0.51 (0.365–0.66)	0.86 (0.77–0.95)	0.74	0.69	0.00015	6.3 (2.4–16)
Irregular or spiculated margin	33/53 (62%)	0.73 (0.60–0.86)	0.64 (0.52–0.77)	0.62	0.75	0.0003	4.95 (2.1–12)
Bulging prostatic contour	31/37 (84%)	0.69 (0.55–0.82)	0.89 (0.81–0.97)	0.80	0.848	< 0.0001	18.5 (6.4–53)
Asymmetry of neurovascular bundle	11/13 (85%)	0.24 (0.12–0.37)	0.96 (0.92–1)	0.85	0.61	0.007	8.7 (1.8–42)
Measurable extraprostatic disease	9/9 (100%)	0.2 (0.08–0.32)	1	1	0.61	0.98	n.d.
Obliteration of recto-prostatic angle	4/4 (100%)	0.09 (0.006–0.17)	1	1	0.58	0.98	n.d.

*mEPE*, suspicion of extraprostatic extension at MRI; *BLR*, binary logistic regression; *PPV*, positive predictive value; *NPV*, negative predictive value; *n.d.*, not determined, interval extending from 0 to infinite

These results were implemented in a comprehensive grading system, labeled mEPE-score (mpMRI-derived extra prostatic extension score), based on the following scores:

- Absence of all mEPE features: 1
- Abutment of the capsule: 2
- Irregular or spiculated margin: 3
- Bulging prostatic contour, asymmetry of the neurovascular bundles, or tumor-capsule interface > 1.0 cm: 4
- At least two of the previous three parameters: 5
- Measurable extraprostatic disease (i.e., obliteration of the recto-prostatic angle or breach of the capsule with evidence of direct tumor extension or bladder wall invasion): 5.

**Fig. 3:**
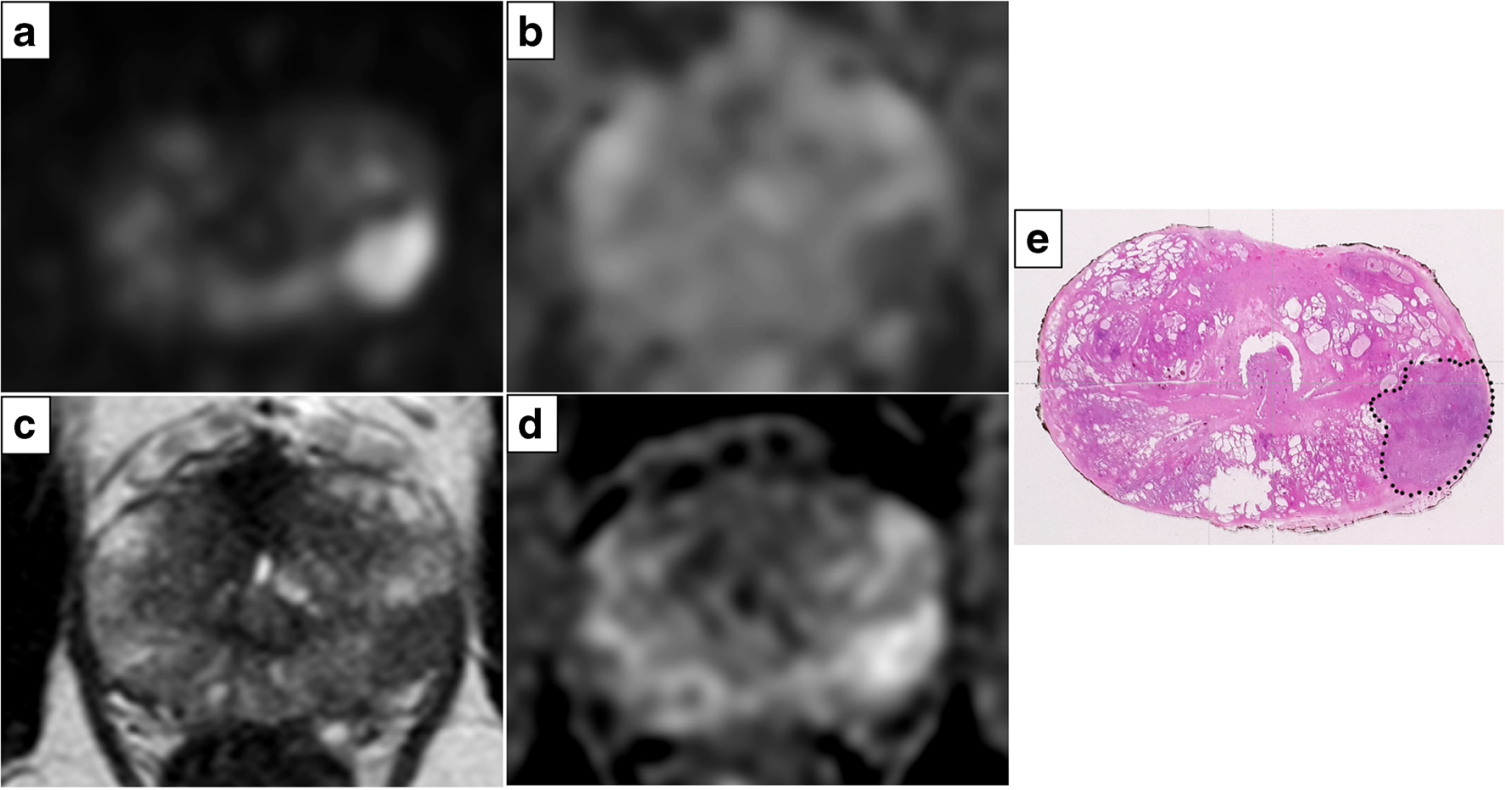
High *b* value (1700 s/mm^2^) DW image (**a**), ADC map (**b**), axial T2-weighted MR image (**c**), and DCE image (**d**). The images showed a lesion in the left peripheral postero-lateral zone in the midportion of the prostate; the tumor-capsule interface was 9 mm, and capsular irregularity was present. The mEPE-score value was 3. In **e**, the histology of a comparable level whole section is presented: a Gleason 4+3 prostate cancer without extraprostatic extension (dotted line)

**Fig. 4:**
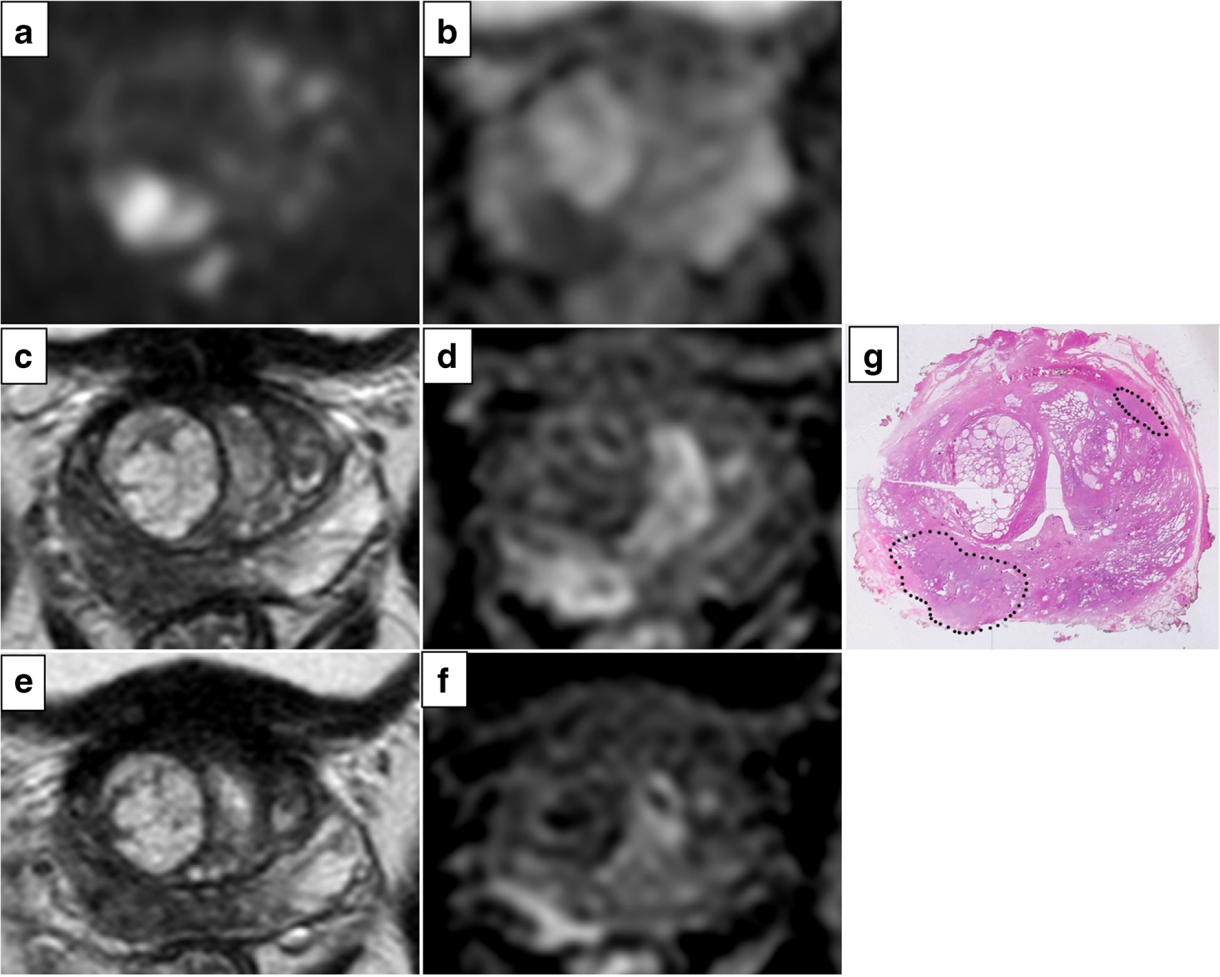
High *b* value (1700 s/mm^2^) DW image (**a**), ADC map (**b**), axial T2-weighted MR image (**c**, **e**), and DCE image (**d**, **f**). The images showed a lesion in the right peripheral postero-medial zone in the midportion of the prostate; the tumor-capsule interface was 21 mm, and capsular irregularity and asymmetry of the neurovascular bundles were present. The mEPE-score value was 5. In **g**, the histology of a comparable level whole section is presented: a Gleason 4+4 prostate cancer with extraprostatic extension (dotted line)

**Fig. 5:**
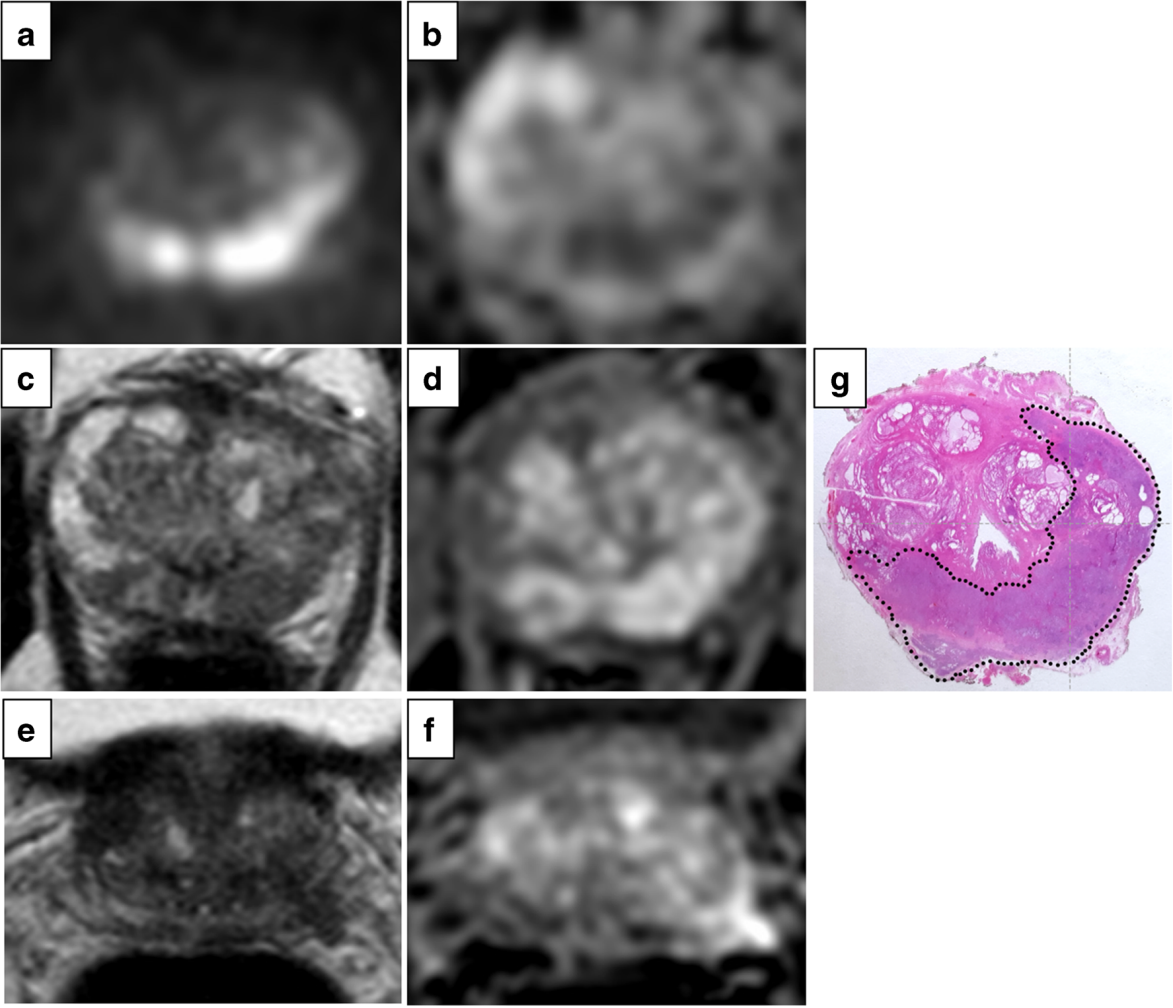
High *b* value (1700 s/mm^2^) DW image (**a**), ADC map (**b**), axial T2-weighted MR image (**c**, **e**), and DCE image (**d**, **f**). The images showed a lesion in the left peripheral postero-medial and lateral and right postero-medial zone in the midportion of the prostate; the tumor-capsule interface was 40 mm, and breach of the capsule with evidence of direct tumor extension outside the capsule was present. The mEPE-score value was 5. In **g**, histology of a comparable level whole section is presented: a Gleason 5+5 prostate cancer with extraprostatic extension (dotted line)

The mEPE-score thus has five possible values: 1 (no indicators), 2, 3, 4, and 5. Examples of tumors classified according to the mEPE-score system are shown in Figs. 3, 4, and 5 and S1.

The accuracy of the proposed score was tested with the ROC curve procedure, obtaining AUC = 0.84 (0.77–0.92). The diagnostic parameters vary with the threshold chosen for pEPE prediction. The three statistical demands agree on mEPE-score > 3 as threshold for positive diagnosis, corresponding to Se = 0.82, Sp = 0.77, PPV = 0.74, and NPV = 0.84. Whenever clinical reasons may require privileging Se over Sp, or vice versa, the threshold might be lowered to > 1 (Se = 0.87, Sp = 0.57, PPV = 0.62, NPV = 0.84) or raised to > 4 (Se = 0.51, Sp = 0.96, PPV = 0.92, NPV = 0.71).

Univariate BLR assessed the significant association of increasing values of mEPE-score with pEPE: *p* < 0.0001, OR = 2.4 (1.7–3.4).

To investigate whether the grade of the disease affected the predicting power of mEPE-score, the 100 patients were subdivided into two groups identified by values of biopsy ISUP < 3 (*N* = 40) and ≥ 3 (*N* = 60). The two respective ROC curves evidenced no significant differences: AUC = 0.80 (0.60–0.99) for ISUP < 3 and 0.82 (0.72–0.92) for ISUP ≥ 3 (*p* = 0.87). The agreement was confirmed by the overlapping of the 95%CIs of the BLR odds ratio for the two ISUP ranges, respectively (2.25 (1.1–4.5) and 2.2 (1.4–3.5)).

### Comparison with other scoring systems

**Fig. 6:**
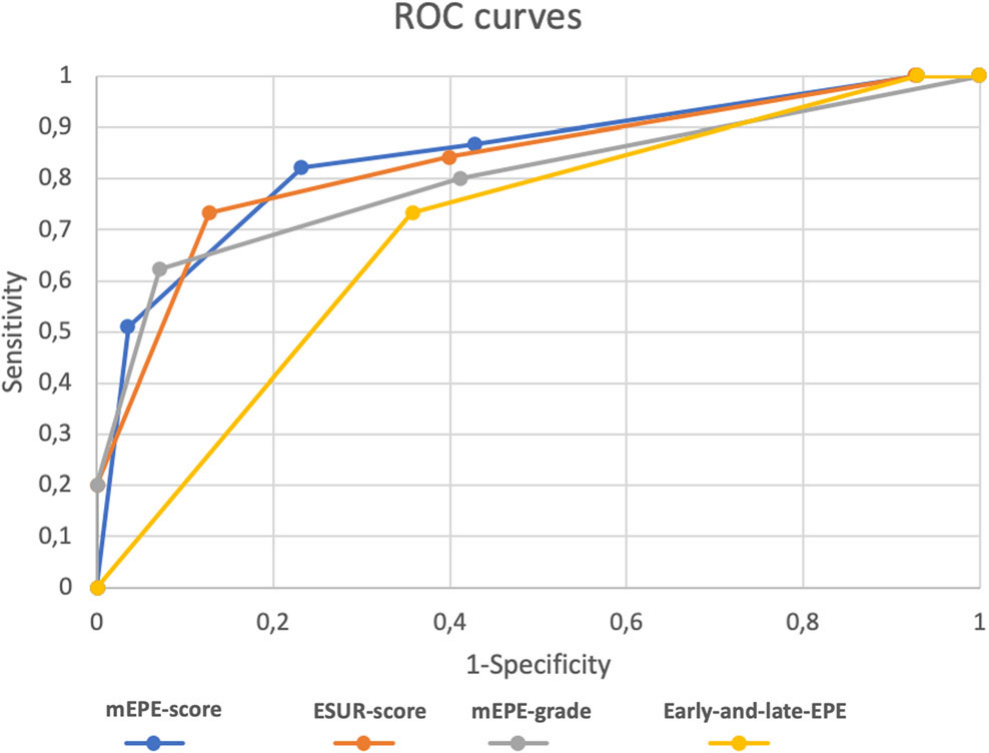
ROC curves of the four quantitative scoring systems

The performance of mEPE-score was tested against those of the three quantitative scoring systems ESUR-score [10], mEPE-grade [11], and early-and-late-EPE [12]. Table 4 and Fig. 6 report the results on the ROC curve procedure: mEPE-score had AUC comparable to ESUR-score (*p* = 0.59) and higher than mEPE-grade (*p* = 0.04) and early-and-late-EPE (*p* < 0.0001). Since the four methods have different score ranges and threshold, we tested the concordance of the prediction on mEPE (yes/no) for each patient with the relative pEPE. The results on Cohen’s *k* are shown at the bottom of Table 4.

**Table 4:** Performance of mEPE-score, ESUR-score [10] mEPE-grade [11] and early-and-late-EPE [12]

	mEPE-score	ESUR-score	mEPE-grade	Early-and-late-EPE
AUC	0.84 (0.77–0.92)	0.83 (0.76–0.91)	0.80 (0.71–0.89)	0.70 (0.61–0.79)
Threshold	> 3	> 3	> 1	> 1
Sensitivity	0.82 (0.71–0.93)	0.73 (0.60–0.86)	0.62 (0.48–0.76)	0.73 (0.60–0.86)
Specificity	0.77 (0.66–0.88)	0.87 (0.78–0.96)	0.93 (0.86–0.99)	0.64 (0.52–0.77)
Accuracy	0.79	0.81	0.79	0.68
PPV	0.74	0.825	0.875	0.62
NPV	0.84	0.80	0.75	0.75
	EPE-score > 3	ESUR-score > 3	EPE-grade > 1	Early-and-late-EPE > 1
Cohen’s kappa	0.60 (0.44–0.76)	0.61 (0.45–0.77)	0.58 (0.42–0.74)	0.38 (0.21–0.56)

*EPE*, extraprostatic extension; *AUC*, area under the curve; *PPV*, positive predictive value; *NPV*, negative predictive value

### Internal validation of EPE-score

The internal validation of mEPE-score was done on the 100 patients of group B. The ROC curve for group B had AUC 0.86 (0.79–0.94), very close to the value 0.84 (0.77–0.92) for group A (*p* = 0.78). The diagnostic parameters at mEPE-score > 3 were Se = 0.80, Sp = 0.78, A = 0.79, PPV = 0.72, and NPV = 0.85.

The univariate BLR yielded *p* < 0.0001, with OR = 2.7 (1.8–4), again very close to the OR = 2.4 (1.7–3.4) obtained for group A. The concordance on each patient between pEPE and mEPE-RADs > 3, measured by Cohen’s *k *= 0.57 (0.41–0.73), was in moderate to substantial agreement with the value of *k* = 0.60 (0.44–0.76) obtained for group A.

### External validation of EPE-score and impact of reader’s experience on the assessment of individual indicator

The sample of 76 patients enrolled at site 2 was used for the external validation of the mEPE-score and for testing the impact of the experience of the mpMRI reader (expressed as number of cases examined) on the identification of the indicators used in the scoring system.

Table 5 shows separately the inter-reader concordance, measured by Cohen’s kappa, of expert reader (> 4000 cases) and beginner reader (> 500) on (i) the individual mEPE features (lowest for capsular abutment and asymmetry of neurovascular bundle and highest for measurable extraprostatic disease), (ii) the score values computed for the four scoring approaches, and (iii) the positive EPE diagnosis (lowest for early-and-late-EPE, similar for mEPE-score, ESUR-score, and mEPE-grade, despite the difference in the value range and threshold). Overall, the percentage agreement between the two readers was 64.5% for the individual features and 88.2% for the positive EPE diagnosis.

**Table 5:** Inter-reader concordance on individual indicators used for the construction of the score

		Cohen’s kappa
Individual features	Capsular abutment	0.34 (0.07–0.61)
	Tumor-capsule interface > 10 mm	0.44 (0.24–0.64)
	Irregular or spiculated margin	0.59 (0.37–0.82)
	Bulging prostatic contour	0.59 (0.41–0.77)
	Asymmetry of neurovascular bundle	0.34 (0.005–0.67)
	Measurable extraprostatic disease	0.84 (0.63–1)
Scoring approaches	mEPE-score	
	Score Positive diagnosis (> 3)	0.61 (0.46–0.75) 0.65 (0.46–0.84)
	ESUR-score	
	Score Positive diagnosis (> 3)	0.70 (0.58–0.81) 0.66 (0.49–0.84)
	mEPE-grade	
	Score Positive diagnosis (> 1)	0.73 (0.60–0.86) 0.68 (0.50–0.86)
	Early-and-late-EPE	
	Score Positive diagnosis (> 1)	0.56 (0.36–0.75) 0.59 (0.38–0.81)

The level of diagnostic performance with mEPE-score, according to the ROC curve procedure, was similar for the two readers: AUC = 0.82 (0.73–0.91) for the expert reader and 0.79 (0.69–0.88) for the beginner reader (*p *= 0.32). Similar agreement was observed for mEPE-grade: AUC = 0.80 (0.72–0.88) for the expert reader and 0.82 (0.74–0.90) for the beginner one (*p* = 0.45), whereas the ESUR-score ROC curves had AUC = 0.83 (0.75–0.90) for the expert reader vs. 0.79 (0.70–0.87) for the beginner reader (*p *= 0.02). A difference was present also for early-and-late-EPE: AUC = 0.63 (0.55–0.71) for the expert reader vs. 0.69 (0.60–0.78) for the beginner one (*p* = 0.03).

The performance of the expert reader with mEPE-score was similar to the performance with mEPE-grade (*p *= 0.58) and ESUR (*p* = 0.82), significantly better than with early-and-late-EPE (*p* = 0.0001).

## Discussion

In our study, each of the PI-RADS v2.1 mEPE feature for predicting pEPE was evaluated for its diagnostic accuracy. A standardized grading system (mEPE-score) was created and tested with internal and external validation. The main findings are that mEPE features have low Se and high Sp and even with tumors of varying aggressiveness and observers’ experience, using a composite score with qualitative and quantitative parameters yielded good diagnostic performances.

As predictors for pEPE, individual PI-RADS v2.1 mEPE features had poor sensitivities ranging from 0.08 (0.00–0.15) to 0.71 (0.59–0.83), whereas Sp ranged from 0.68 (0.58–0.79) to 1.00. Capsule abutment had the highest Se, but low Sp. PI-RADS v2.1 considers a contact of over 10 mm significant for pEPE. This feature in our derivation set had a Se of 0.48 (0.34–0.62) and Sp of 0.89 (0.82–0.96), lower in Se but higher in Sp than those reported in a recent meta-analysis [20], in which the summary Se and Sp were 0.79 (0.73–0.83) and 0.67 (0.60–0.74). Our data still agreed with some of the papers in that study, namely 5 out of 13 studies used to evaluate the capsule tumor contact length’s Se and 4 out of 13 studies’ Sp.

Breach of the capsule with evidence of direct tumor extension and obliteration of the recto-prostatic angle were the most specific parameters. These data are consistent with the findings of Mehralivand et al [11]. Since these features have very high and comparable Sp, and are frequently correlated, they were summarized and evaluated as “measurable extraprostatic disease” in the readings performed at site 2.

A recent meta-analysis [4] of 45 studies and 5681 patients reported a Se of 0.57 (0.49–0.64) and Sp of 0.91 (0.88–0.93) in assessing mEPE. However, there is no universal definition of mEPE; instead, numerous criteria are reported, and the data, expressed in a binary format, can be misleading. The PI-RADS v2.1 document recommends reporting suspicious features for mEPE for staging, but refrains from assigning a probability of pEPE based on a combination of these findings.

The mEPE-score is a score that combines all the parameters included in PI-RADS v2.1 for the assessment of pEPE and showed excellent discriminating ability (i.e., AUC > 0.8 both in the derivation and validation set) [21] and Se = 0.82, Sp = 0.77, PPV = 0.74, and NPV = 0.84 with a threshold of 3 in the derivation set.

The “ESUR prostate MR guidelines 2012” [10] for the first time introduced an increasing probability EPE-score (ESUR-score). However, this score is purely qualitative and does not include the extent of the tumor-capsule interface, a parameter that in the aforementioned meta-analysis [20] showed good diagnostic power.

Mehralivand et al [11] recently introduced mEPE-grade, a system which ranked the risk of pEPE from 1 to 3 and guaranteed an AUC of 0.77 (up to 0.81 when combined with clinical features). However, the tumor-capsule interface considered positive in the mEPE-grade was 15 mm rather than 10 mm, and the capsule abutment and asymmetry of the neurovascular bundle were omitted. This score performed at roughly the same level as our proposed score (worse, albeit by a small margin, *p* = 0.04, in the internal validation and comparable, *p *= 0.58, in the external validation). However, while using a scale with “only” four points produces excellent AUC results, having to dichotomize to find a clinically useful cutoff may cause issues, as shown by the lowest Se (i.e., 0.55 vs. 0.75 of the EPE-score). Increasing the cutoff of capsule tumor contact reduces Se, as does using fewer mEPE features than mEPE-score.

The most recent score published in the literature is that by Pesapane et al [12], who categorize mEPE features into “early” and “late,” with the first having a high prevalence but a low PPV and the latter having a lower prevalence but a higher PPV. However, this score is purely qualitative, and when compared to the results of the single predictor analysis, two features do not match our findings: they included capsule abutment (features with very low Sp) in the “early”; additionally, they included irregular prostatic contour in the “late” (as “measurable extraprostatic disease”), which demonstrated a Sp and positive predictive value of less than 0.7 in our dataset.

The impact of reader’s experience in lesion detection at mpMRI has already been reported [22]. Using an mpMRI-derived EPE-score simplifies the process of reporting pEPE suspicion at mpMRI. This is underlined by the fact that while concordance between observers with varying levels of experience ranged from fair to moderate for each mEPE feature (except for the identification of “measurable extraprostatic disease”), the use of a score resulted in substantial agreement for the majority of scores included in the analysis (except for early-and-late-EPE). Our findings are consistent with those of Pesapane et al [12], who demonstrated that agreement between two readers for “late signs” was significantly greater than that for “early signs,” reaching a score of 0.94 for recto-prostatic angle obliteration and periprostatic mass, and with those of Park et al [14], who reported similar concordance (from 0.63 to 0.71) with the use of mEPE-grade and ESUR-score. Moreover, for mEPE-score, both observers performed comparably, while with the use of ESUR-score and early-and-late-EPE had significant differences. This could be at least partly related to the inclusion of a quantitative parameter within the scores.

Clinically, our findings strongly suggest that a score should be used to define mEPE, as this is critical for obtaining consistent and reliable results. Nowadays, mpMRI is the first diagnostic step in the work-up for PCa, and the only data available to the radiologist during the examination are the results of the urological examination and the PSA value. Numerous scores developed for the evaluation of EPE incorporate biopsy data but do not include mpMRI [23, 24]. Due to the excellent performance and wide availability of mpMRI, a purely radiological score may provide radiologists with an initial reliable information to be eventually integrated into the clinical scenario. Moreover, the possibility of tailoring the threshold based on the specific needs of the patient under examination (raising the cutoff to 4 for want of higher specificity or lowering to 2 to increase sensitivity) is a further help for the urologist. Overall, some attempts have already been made in this direction [11, 25–27] and the work presented here aspires to be the foundation for standardizing the evaluation of mEPE and generating a comprehensive highly performing clinical-radiological score.

This study has some limitation. First, it was a retrospective study, albeit one of the largest and from two Institutions. Second, the mpMRIs of each site were evaluated by radiologists of the local staff, so we cannot exclude performance disparities. However, we believe that this option was preferable than having a single radiologist interpreting also exams obtained from MRI scanners which she/he was unfamiliar with. Third, we tested the different scores only on examinations done without endorectal coil and on a single vendor MRI scanner.

In conclusion, even if the individual mEPE features used to assess pEPE in PI-RADS v2.1 have low Se and high Sp, the use of mEPE-score, a score that includes all of them, allows for consistent and reliable assessment for pEPE.

## Supplementary Information

Figure 1s. High-b-value (1700 s/mm2) DW image (A), ADC map (B), Axial T2-weighted MR image (C), DCE image (D). The images showed a lesion in the right peripheral postero-lateral zone in the midportion of the prostate; the tumor-capsule interface was 6 mm and no other mEPE features was present. The mEPE-score value was 2. In E the histology of a comparable level whole section is presented: a Gleason 4+3 prostate cancer without extraprostatic extension (dotted line). (PNG 1124 kb)

ESM 1 (DOCX 19 kb)

## Abbreviations

AUC: Area under the curve
BLR: Binary logistic regression
ESUR: European Society of Urogenital Radiology
mEPE: Suspicion of extraprostatic extension at MRI
mEPE-score: mpMRI-derived extra prostatic extension score
mpMRI: Multiparametric magnetic resonance imaging
NPV: Negative predictive value
PCa: Prostate cancer
pEPE: Pathologic extra prostatic extension
PI-RADS: Prostate Imaging Reporting and Data System
PPV: Positive predictive value
ROC: Receiver operating characteristic
Se: Sensitivity
Sp: Specificity

## Declarations

### Guarantor

The scientific guarantor of this publication is Prof. Paolo Fonio.

### Statistics and biometry

One of the authors has significant statistical expertise.

### Methodology

• retrospective

• diagnostic or prognostic study

• multicenter study
