# Supplementary figures and images for: mEPE-score: a comprehensive grading system for predicting pathologic extraprostatic extension of prostate cancer at multiparametric magnetic resonance imaging
Source: Eur Radiol. 2022 Mar 15;32(7):4942–53. doi: 10.1007/s00330-022-08595-9 (PMC9213375; doi:10.1007/s00330-022-08595-9)

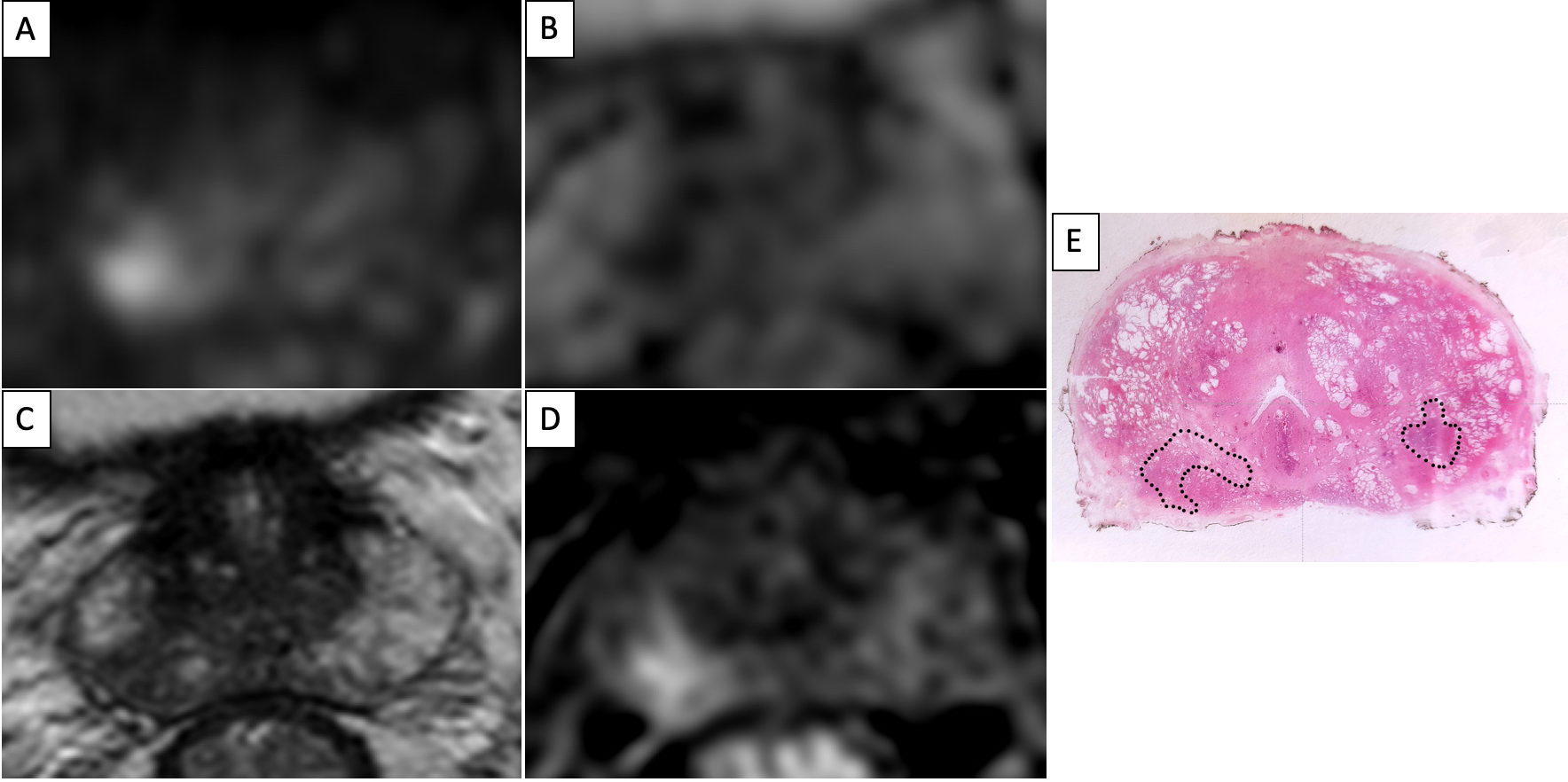

Supplement: Supplementary file 1 — High-b-value (1700 s/mm2) DW image (A), ADC map (B), Axial T2-weighted MR image (C), DCE image (D). The images showed a lesion in the right peripheral postero-lateral zone in the midportion of the prostate; the tumor-capsule interface was 6 mm and no other mEPE features was present. The mEPE-score value was 2. In E the histology of a comparable level whole section is presented: a Gleason 4+3 prostate cancer without extraprostatic extension (dotted line). (PNG 1124 kb) [file 330_2022_8595_MOESM1_ESM.png]
