# Supplementary material for: mEPE-score: a comprehensive grading system for predicting pathologic extraprostatic extension of prostate cancer at multiparametric magnetic resonance imaging
Source: Eur Radiol. 2022 Mar 15;32(7):4942–53. doi: 10.1007/s00330-022-08595-9 (PMC9213375; doi:10.1007/s00330-022-08595-9)
Supplement: Supplementary file 2 — (DOCX 19 kb) [file 330_2022_8595_MOESM2_ESM.docx]

***Table 1s.*** *Technical parameters of the Site 1 acquisition protocol (1.5 T scanner, Achieva, Philips Medical Systems, Eindhoven, The Netherlands).*

| **Parameters** | **T2w axial** | **T2w sagittal** | **T2W coronal** | **DWI** | **DCE** |
| --- | --- | --- | --- | --- | --- |
| **Field of view (mm)** | 180 x 180 | 180 x 180 | 180 x 180 | 180 x 180 | 180 x 180 |
| **In plane resolution (mm)** | 0.38 x 0.38 | 0.47 x 0.47 | 0.47 x 0.47 | 1.88 x 1.88 | 0.94 x 0.94 |
| **section thickness/gap (mm)** | 3/0 | 3/0 | 3/0 | 3/0 | 3/0 |
| **Repetition time (ms)** | 5403 | 5214 | 4606 | 2919 | 4.7 |
| **Echo time (ms)** | 100 | 100 | 100 | 61 | 2.2 |
| **Flip angle (degrees)** | 90 | 90 | 90 | 90 | 10 |
| **b Values (s/mm2)** | n.a. | n.a. | n.a. | 50,500,1000 and separate 1700 | n.a. |

***Table 2s.*** *Technical parameters of the Site 2 acquisition protocol (1.5 T scanner, Ingenia, Philips Medical Systems, Eindhoven, The Netherlands).*

| **Parameters** | **T2w axial** | **T2w sagittal** | **T2W coronal** | **DWI** | **DCE** |
| --- | --- | --- | --- | --- | --- |
| **Field of view (mm)** | 140 x 140 | 180 x 180 | 140 x 140 | 250 x 250 | 240 x 240 |
| **In plane resolution (mm)** | 0.36 x 0.36 | 0.31 x 0.31 | 0.36 x 0.36 | 2.23 x 2.23 | 0.71 x 0.71 |
| **section thickness/gap (mm)** | 3/0 | 3/0 | 3/0 | 3/0 | 3/0 |
| **Repetition time (ms)** | 3356 | 6283 | 3712 | 4061 | 4.9 |
| **Echo time (ms)** | 120 | 120 | 120 | 74 | 2.3 |
| **Flip angle (degrees)** | 90 | 90 | 90 | 90 | 10 |
| **b Values (s/mm2)** | n.a. | n.a. | n.a. | 0,500,1000,1500 and separate 2000 | n.a. |
